# Cost‐effective purification process development for chimeric hepatitis B core (HBc) virus‐like particles assisted by molecular dynamic simulation

**DOI:** 10.1002/elsc.202000104

**Published:** 2021-05-03

**Authors:** Bingyang Zhang, Shuang Yin, Yingli Wang, Zhiguo Su, Jingxiu Bi

**Affiliations:** ^1^ School of Chemical Engineering & Advanced Materials, Faculty of Engineering, Computer and Mathematical Sciences University of Adelaide Adelaide SA Australia; ^2^ School of Chinese Medicine and Food Engineering Shanxi University of Traditional Chinese Medicine Jinzhong Shanxi Province P. R. China; ^3^ State Key Laboratory of Biochemical Engineering, Institute of Process Engineering Chinese Academy of Sciences Beijing P. R. China

**Keywords:** hepatitis B core, molecular dynamic simulation, protein characterization, protein purification, virus‐like particle

## Abstract

Inserting foreign epitopes to hepatitis B core (HBc) virus‐like particles (VLPs) could influence the molecular conformation and therefore vary the purification process. In this study, a cost‐effective purification process was developed for two chimeric HBc VLPs displaying Epstein–Barr nuclear antigens 1 (EBNA1), and hepatitis C virus (HCV) core. Both chimeric VLPs were expressed in soluble form with high production yields in *Escherichia coli*. Molecular dynamic (MD) simulation was employed to predict the stability of chimeric VLPs. HCV core‐HBc was found to be less stable in water environment compared with EBNA1‐HBc, indicating its higher hydrophobicity. Assisting with MD simulation, ammonium sulfate precipitation was optimized to remove host cell proteins with high target protein recovery yields. Moreover, 99% DNA impurities were removed using POROS 50 HQ chromatography. In characterization measurement, we found that inserting HCV core epitope would reduce the ratio of α‐helix of HCV core‐HBc. This could be another reason on the top of its higher hydrophobicity predicted by MD simulation, causing its less stability. Tertiary structure, transmission electron microscopy, and immunogenicity results indicate that two chimeric VLPs maintained correct VLP structure ensuring its bioactivity after being processed by the developed cost‐effective purification approach.

**Abbreviations**
AEC: anionic exchange chromatography
ARD: arginine‐rich domain
AS: ammonium sulfate
CD: circular dichroism
*E. coli*: *Escherichia coli*
EBNA1: Epstein–Barr nuclear antigen 1
ELISA: enzyme‐linked immunosorbent assay
FT: flow‐through
HBc: hepatitis B core
HCP: host cell protein
HCV core: hepatitis C virus core
HPSEC: high‐performance size‐exclusion chromatography
IF: intrinsic fluorescence
LB: Luria–Bertani
MALLS: multi‐angle static laser light scattering
MD: molecular dynamic
PB: phosphate buffer
PCR: polymerase chain reaction
Rg: radius of gyration
RMSD: root‐mean‐square deviation
SDS‐PAGE: sodium dodecyl sulfate‐polyacrylamide gel electrophoresis
TEM: transmission electron microscopy
VLP: virus‐like particle

## 1 INTRODUCTION

Chimeric virus‐like particle (VLP)‐based vaccine is a novel technology that generates vaccine candidates against oncoviruses that potentially lead to cancers [1]. To expand the application of VLP‐based vaccines, chimeric VLPs, which were constructed by fusing foreign epitopes or antigens to well‐studied VLPs, have been developed for the treatment of several viral infection diseases such as influenza [2, 3] and cancers [4, 5, 6]. Hepatitis B core (HBc) VLP is reported to be one of the most powerful VLP candidates to (1) display foreign epitopes for specific immunogenicity [7, 8], (2) expose cell‐targeting signals [9, 10], and (3) package poly‐ and oligonucleotides [11]. Comparing with chemical coupling, antigens added through genetic modification is more controllable on, particularly, the antigen density and the insertion position. These modifications significantly influence the immunogenicity of chimeric VLPs. However, challenges of removal of host cell proteins (HCPs) and host cell DNA remain for the fused VLP proteins in their purification process. Therefore, rapid, efficient, and cost‐effective production process of chimeric HBc VLPs is needed for large scale production and future commercialization.

Previous works have proven that HBc VLP is able to display various foreign epitopes including HCC epitopes: MAGE‐1, MAGE‐3, AFP1, and AFP2 [12], B cell epitopes of foot‐and‐mouth disease virus [13], and SP55 and SP70 epitope from EV71 [14]. However, challenges remained, such as performing effective expression of chimeric HBc VLPs, removal of impurities, and maintenance of assembled VLP structure after purification. In addition, easily formation of mis‐folding and aggregation was found after insertion of epitope to HBc VLP [15, 16]. These challenges can lead to failure to induce epitope‐specific immune response [17].

To perform an effective expression of chimeric HBc VLPs, prokaryotic expression system such as bacteria expression system is highly regarded because of economical process and high expression yield [18, 19]. The higher production yield and shorter production time of VLPs make bacteria expression system stand out of other expression system such as mammalian cells and insect cells [20]. However, reports have indicated that VLPs expressed by bacteria expression system may be expressed as insoluble form, namely inclusion bodies (IBs). This phenomenon increases the complexity in the downstream purification because a refolding process is required [13, 14, 21]. To improve the soluble expression of chimeric VLP proteins in bacteria expression system, expression conditions including temperature, inducer concentration, and density of bacteria for expression have been controlled during the expression process [22].

**PRACTICAL APPLICATION**

This work aims to develop a cost‐effective purification process for two chimeric hepatitis B core (HBc) virus‐like particles (VLPs) with the insertion of epitopes against Epstein–Barr virus and hepatitis C virus (HCV) infection. Impacts of two epitopes on production of chimeric HBc VLPs were investigated. Insertion of long and structural HCV core epitope was found to have a significant impact on the conformation, and it increased the complexity of purification process, compared with short and non‐structural epitope. Inspired by the prediction of chimeric protein stability using molecular dynamic simulation, cost‐effective purification process has been established using ammonium sulfate precipitation and chromatography to remove host cell proteins and DNA with high production yield and purity. Characterization results confirmed that both produced chimeric HBc VLPs were in correctly assembled VLP structure that can ensure their desired antigenicity. Therefore, this purification process is suitable for scalable production and further commercialization.

To meet the regulation requirement of biopharmaceutical products regarding safety and efficacy, effective downstream purification process is crucial to remove the impurities, including HCPs and host cell DNA. Different from other recombinant proteins, purification of VLP proteins has the challenges to maintain stable VLP structure, which is directly related to its immunogenicity after the purification process [23, 24]. To remove HCPs, various purification methods have been developed based on the properties of VLPs such as molecular weight, size, surface charge, and hydrophobicity. For example, ultracentrifugation was applied based on molecular weight difference. However, the yield of this approach is relatively low, and the capital cost is relatively high and challenged in high‐throughput process. Chromatography is another popular approach based on difference of hydrophobicity, charge density, molecular size [25, 26, 27, 28]. For example, Butyl‐S Sepharose 6FF chromatography and Superdex 200 chromatography were applied for the removal of HCPs for HBc VLP [27]. High purity was achieved, however, after multistep purification process including heat‐treatment, hydrophobic interaction chromatography, ultrafiltration, and size‐exclusion chromatography, the final recovery yield was 41.92% indicating a large production loss. In addition, Yang et al. reported that changes in microenvironments such as pH and ion strength can affect the interactions between VLPs and column resin, and harsh operating conditions can lead to the aggregation or disassembly of target VLPs [29]. Because ammonium sulfate (AS) is a non‐inactivating protein precipitant and stabilizer that has a minor impact on the VLP structure, low‐cost precipitation has been applied in the purification of HBc VLPs [30] and HPV VLPs [31] by changing protein solubility [32]. However, the optimal condition (pH, temperature, and AS concentration) for AS precipitation varies according to molecule conformation changed caused by the inserted epitope [33].

Among different approaches to remove host cell DNA, flow‐through strategy using anionic exchange chromatography (AEC) to bind nucleic acid impurities has been reported as an efficient approach [34]. After comparing several different anionic resins including POROS 50 HQ, Mustang Q, Sartobind Q Nano, Fractogel TMAE, and Q Sepharose Fast Flow, POROS 50 HQ achieved significantly higher dynamic DNA binding capacity over a range of processing conditions, including tolerance of high salt concentration [35], which makes it more efficient and cost‐effective. In addition, our research group has also developed an efficient approach to dissociate protein‐DNA complexes using optimized salt concentration and then remove the DNA impurities from host cells using chromatography [36].

To further study the impact of insertion of foreign epitopes to HBc sequence on the conformation and the stability of chimeric HBc VLPs, molecular dynamic (MD) simulation technique has been developed to analyze physical movements of atoms and molecules of target VLP have been developed. For example, root‐mean‐square deviation (RMSD) and radius of gyration (Rg) have been applied in the study of the stability of proteins [37, 38]. It is regarded that the smaller the value of RMSD and lower fluctuation level of Rg are the more stable target protein is. This can guide the design of the purification process of chimeric HBc VLPs and save massive experimental time.

In this study, we aim to develop a cost‐effective and efficient production process for the chimeric HBc VLPs with high purity and high production yield. Two different epitopes, Epstein–Barr nuclear antigens 1 (EBNA1), and hepatitis C virus (HCV) core, were selected to fuse to the N‐terminus of HBc VLP to form chimeric EBNA1‐HBc VLP and HCV core‐HBc VLP. EBNA1 and HCV core were reported as two epitopes targeting to Epstein–Barr virus and HCV infection, respectively, leading to cancers. EBNA1 epitope was chosen as the representative of short and non‐structural epitope while HCV core was chosen as long and structural epitope for the study of impact of insertion of different epitopes on the purification of chimeric HBC VLPs. Chimeric HBc VLPs were expressed using *Escherichia coli* expression system. Fermentation condition optimization was conducted to improve HCV core‐HBc soluble expression level. Computational analysis was adopted to predict the stability of chimeric HBc‐VLPs. After expression, AS precipitation was applied for the removal of HCPs and compared between two chimeric HBc VLPs. Host cell DNA of chimeric HBc VLP proteins were then removed using POROS 50 HQ chromatography. Characterization of purified HCV core‐HBc VLP and EBNA1‐HBc VLP were examined using different approaches including sodium dodecyl sulfate‐polyacrylamide gel electrophoresis (SDS‐PAGE), high‐performance size‐exclusion chromatography (HPSEC)‐multi‐angle static laser light scattering (MALLS), intrinsic fluorescence (IF) and transmission electron microscopy (TEM) compared with standard HBc VLP to evaluate the structure of the target chimeric VLPs.

## 2 MATERIALS AND METHODS

### 2.1 Materials

pET 21a(+)‐HBc plasmid was obtained from State Key Laboratory of Biochemical Engineering of Institute of Process Engineering (IPE) of Chinese Academy of Sciences (CAS) (China) and pET 30a‐EBNA1‐HBc was purchased from Beijing Genomics Institute (China). Tryptone, yeast extract, isopropyl β‐D‐thiogalactoside (IPTG) and kanamycin were purchased from Thermo Scientific (USA). POROS 50 HQ was purchased from Thermo Fisher Scientific (USA) and TSKgel G4000SWXL column was purchased from TOSOH Bioscience (Japan). Chromatography columns were bought from Cytiva (USA).

All other reagents were analytical grade, and solutions were prepared using Milli‐Q water (Millipore, USA). Chromatography was performed on ÄKTA pure system from Cytiva (USA).

### 2.2 Plasmids for recombinant HCV core‐HBc and EBNA1‐HBc

The HBc gene in plasmid pET 21a(+)‐HBc (State Key Laboratory of Biochemical Engineering of IPE of CAS, China) was released by XhoI/NdeI digestion and then ligated into pET 30a plasmid (IPE of CAS, China) to generate an intermediate plasmid pET 30a‐HBc. To make HCV core (aa 10–53: KTKRNTNRRPQDVKFPGGGQIVGGVYLLPR RGPRLGVRATRKTS) fused HBc protein, two step of polymerase chain reactions (PCRs) were performed. Briefly, in the first step, two complementary oligonucleotides HCV core‐F, GGAATTCCATATGAAAACCAAAAGAAACACA and HCV core‐R, ACCGAACTCTTT GTATGGGTCGATGTCCATACT were designed and used to amplify the HCV core sequence from the template supplied from Beijing Genomics Institute, China. Then another two complementary oligonucleotides HBc‐F, ATGGACATCGACCCATACAAAGAGTTC GGT and HBc‐R, CCGCTCGAGTTA ACACTGAGATTCACG were applied to amplify the HBc sequence from pET 30a‐HBc template. In the second step, HCV core‐F, GGAATTCCATATG AAAACCAAAAGAAACACA and HBc‐R, CCGCTCGAGTTAACACTGAGATTCACG were used for an overlapped PCR to obtain HCV core‐HBc gene sequence. The obtained HCV core‐HBc gene sequence was then ligated into pET 30a plasmid to yield pET 30a‐HCV core‐HBc plasmid. Plasmid pET 30a‐EBNA1 (aa 407–417: HPVGEADYFEY)‐HBc was supplied by Beijing Genomics Institute, China. The obtained plasmid sequence was confirmed by gene sequencing by Beijing Genomics Institute, China.

### 2.3 Expression of chimeric HBc VLPs in *E. coli* expression system

After confirming the plasmid sequences of chimeric HBc VLPs, the plasmids were transferred to *E. coli* BL21 (DE3) strain (Thermo Fisher Scientific, USA) for the expression in Erlenmeyer flask in the shaker. pET 30a‐HCV core‐HBc and pET EBNA1‐HBc were transferred to *E. coli* competent cells, which were grown on Luria–Bertani (LB) agarose plate overnight at 37°C. Then, a single colony of cell that containing HCV core‐HBc or EBNA1‐HBc was cultured in 5 ml of LB medium (Thermo Fisher Scientific, USA) supplemented with 100 μg/mL kanamycin (Thermo Fisher Scientific, USA) at 37°C overnight, respectively. Fifty microliters of pre‐cultured chimeric HBc BL21 *E. coli* was cultured in 50 ml LB medium supplemented with 100 μg/mL kanamycin at 37°C overnight. *E. coli* BL21 cells containing HCV core‐HBc and EBNA1‐HBc plasmids were stored in 25% glycerol at −70°C for future use. Initially, the expression conditions of HCV core‐HBc and EBNA1‐HBc followed the previous work of HBc VLP [39]. Briefly, the pre‐cultured chimeric HBc *E. coli* cells were transferred to 2 L of LB medium with 100 μg/mL kanamycin in the ratio of 1:1000 (v/v) and the cultivation was cultured at 37.5°C for 4 h at 220 rpm. One millimolar of IPTG (Thermo Fisher Scientific, USA) was added for the expression of HCV core‐HBc and EBNA1‐HBc proteins. The culture was then incubated for 4 h at 37°C with 180 rpm.

To improve the soluble expression of HCV core‐HBc, single‐factor optimization of expression conditions was conducted by using 0.8 for the induction cell density of bacteria, 30°C for expression temperature, and 0.5 mM for the concentration of IPTG.

*E. coli* cells after expression were then harvested by centrifugation at 4000 rpm for 20 min at 4°C. The pellets were resuspended with 100 ml lysate buffer (20 mM Tris‐HCl, 3 mM EDTA, 1 mM phenylmethanesulfonylfluoride, 0.1% Triton X100, pH 8.0). Ultrasonic homogenizer (Scientz‐IID, Ningbo Scientz Bio‐Tech Co, Ltd, China) with a 4‐s on and 6‐s off pulse was used for the disruption of *E. coli* cells for 10 min at 360 W. The crude lysate was then centrifuged at 10,000 rpm for 30 min at 4°C. The supernatant and pellets after centrifugation together with the crude lysate before centrifugation were loaded to 12% reducing SDS‐PAGE for evaluation later. The concentrations of total soluble proteins of chimeric HBc VLPs (c_total soluble proteins_) were examined using Bradford assay [40].The target protein concentration in SDS‐PAGE (c_target protein_) was estimated using ImageJ [41] and percentage of soluble target protein in the supernatant of bacteria lysate was calculated using Equation (1):

(1) $$\begin{array}{ccc} & & Soluble\;target\;protein\;\;\left(\%\right)\\ & & \;=\frac{c_{target\;protein\;in\;supernatant\;of\;bacteria\;lysate}}{c_{total\;taregt\;protein\;in\;crude\;lysate}}\;\\ & & \;\times \;100\% \end{array}$$

### 2.4 AS precipitation of chimeric HBc VLPs

AS precipitation was applied to purify HCV core‐HBc and EBNA1‐HBc proteins because of its minor influence on their VLP structure. The purity and recovery yield were expected to be high. AS precipitation condition for HBc VLP in previous work [39] was applied for purification of HCV core‐HBc and EBNA1‐HBc proteins. Briefly, 1 M AS was added to the crude lysate of HCV core‐HBc and EBNA1‐HBc samples. Then the mixture was centrifuged at 6000 rpm for 10 min. The mixture was stirred for 30 min at 25°C. The pellet was resuspended using 4 M urea buffer (50 mM glycine‐NaOH with 4 M urea, pH 9). Resuspended samples were centrifuged at 10,000 rpm for 30 min.

To improve the recovery yield of AS precipitation for HCV core‐HBc, different concentrations of AS including 1, 0.5, 0.1, 0.05, and 0.01 M were examined. The pellets of each condition were then collected and resuspended using 4 M urea buffer (50 mM glycine‐NaOH with 4 M urea, pH 9). Resuspended samples were centrifuged at 10,000 rpm for 30 min. After purification using AS precipitation, both purified EBNA1‐HBc and HCV core‐HBc proteins were dialyzed against storage buffer (20 mM Tris‐HCl buffer, pH 7.4). Supernatant samples after precipitation, supernatant samples after resuspension, pellet samples after resuspension, and supernatant samples after dialysis were loaded to 12% reducing SDS‐PAGE for examination. The concentrations of the supernatant of resuspended chimeric HBc VLPs and supernatant of chimeric HBc VLPs after dialysis were measured using Bradford assay [40]. The purity of target protein was estimated according to the intensity calculation using SDS‐PAGE image using ImageJ [41] and recovery yield of AS precipitation was calculated using Equation (2):

(2) $$\begin{array}{ccc} & & AS\;precipitation\;revovery\;yield\;\\ & & \;=\frac{c_{supernatant\;after\;resuspension}\times Purity\;after\;purification\;\left(\%\right)}{c_{total\;soluble\;proteins}\times Purity\;before\;purification\;\left(\%\right)}\;\\ & & \;\times \;100\% \end{array}$$

The purity of target protein was estimated according to the intensity calculation using SDS‐PAGE image using ImageJ and the production yields of chimeric HBc VLPs were calculated using Equation (3):

(3) $$\begin{array}{ccc} & & Production\;yield\;\\ & & \;=\frac{c_{supernatatant\;after\;dialysis}\times Purity\;of\;target\;protein\;\left(\%\right)}{mass\;{\left(g\right)}_{wet\;biomass}\;or\;volume{\left(L\right)}_{biomass}}\;\\ & & \;\times \;100\% \end{array}$$

### 2.5 Flow‐through anionic chromatography for chimeric HBc VLPs

Flow‐through strategy using AEC was applied to separate chimeric HBc VLP proteins from binding nucleic acid impurities. Briefly, POROS 50 HQ chromatography (Thermo Fisher Scientific, USA) were applied using ÄKTA pure system (Cytiva, USA). Chimeric HBc VLP samples in storage buffer were loaded to POROS 50 HQ chromatography using 20 mM Tris‐HCl, pH 9 at a flowrate of 1 ml/min and one stepwise elution was performed using 20 mM Tris‐HCl, 1 M NaCl, pH 9. Flow‐through (FT) fractions and elution fractions (P1) of EBNA1‐HBc, HCV core‐HBc, and HBc were loaded to 12% reducing SDS‐PAGE for evaluation. The purity of target protein was estimated using ImageJ [41]. The concentration of proteins and nucleic acids in FT and P1 were examined using Bradford assay [40] and Quant‐iT 1X dsDNA HS Assay kit (Thermo Fisher Scientific, USA). Protein recovery yield was calculated using Equation (4):

(4) $$\begin{array}{ccc} & & Protein\;recovery\;yield\;\\ & & \;=\frac{m_{protein\;in\;FT\;fraction}\times Purity\;of\;target\;protein\;in\;FT}{m_{protein\;before\;loading}\times Purity\;of\;target\;protein\;before\;loading}\;\\ & & \;\times \;100\% \end{array}$$

DNA removal rate was calculated using Equation (5):

(5) $$\begin{array}{ccc} & & DNA\;removal\;rate\;\\ & & \;=\;\left(1-\frac{c_{DNA\;in\;FT\;fraction}}{c_{DNA\;before\;loading\;to\;chromatography}}\right)\\ & & \;\times \;100\% \end{array}$$

### 2.6 Characterization of chimeric EBNA1‐HBc VLP

Characterization of purified EBNA1‐HBc VLP samples were evaluated with SDS‐PAGE, HPSEC‐MALLS, IF, and TEM.

#### 2.6.1 SDS‐PAGE

SDS‐PAGE was used to separate the protein samples according to their molecular weights and to evaluate the purity of target protein in samples [42]. In our SDS‐PAGE analysis, 12% SDS polyacrylamide gel was employed. Protein samples (5 μg per lane) were mixed with SDS‐gel electrophoresis sample loading buffer containing 5% SDS and 5% 2‐mercaptoethanol. Samples were heated at 100°C for 10 min before loading to the SDS‐PAGE gel. Ninety volts of power was used for the stacking gel and 120 V of power was used for the separating gel. The proteins samples were separated on 12% SDS polyacrylamide gel in vertical chambers (Bio‐Rad, USA).

#### 2.6.2 HPSEC‐MALLS

To further detect the molecular weight of the protein samples, MALLS was applied as it measures the light scattered by a sample into a plurality of angles. In our study, the purity and molecular weight of purified EBNA1‐HBc VLP were evaluated by HPSEC‐MALLS. HPSEC analysis was performed in Shimadzu UHPLC XR system (Shimadzu, Japan), equipped with TSKgel G4000SWXL column (TOSOH Bioscience, Japan). Fifty microliters of sample was loaded and eluted at 0.8 ml/min with 50 mM phosphate buffer (PB) with 100 mM sodium sulfate, pH 7.4. Elution buffer was degassed and filtered by 0.22 μm Millipore membrane (Pall Corporation, USA). The retention time and absorbance at 280 nm were recorded. The HPSEC system was coupled to a MALLS Wyatt DAWN HELEOS II and Optilab T‐rEx (Wyatt Technology, USA) for the detection of the molecular weight. The data were processed with ASTRA software (v. 6.1).

#### 2.6.3 CD spectroscopy

Circular dichroism (CD) is an absorption spectroscopy method based on the differential absorption of left and right circularly polarized light and is commonly employed in the evaluation of the secondary structure of protein samples [43]. In our study, difference of secondary structures between EBNA1‐HBc VLP and HBc VLP was detected by a Jasco J‐810 spectropolarimeter (Jasco, Japan), using a quartz cuvette with 0.1 cm pathlength. All the protein samples were prepared at the concentration of 0.4 mg/mL in 20 mM PB (pH 7.4). The baseline of buffer was subtracted from the experimental spectra for corrections. The reported CD spectra are the average of three scans.

#### 2.6.4 IF

IF is a type of electromagnetic spectroscopy using ultraviolet light to excite the electrons in molecules of proteins [44]. IF spectroscopy can be applied to evaluate the conformational state of protein samples due to the tryptophan fluorescence shifts [45]. HBc VLP and EBNA1‐HBc VLP were analyzed using Hitachi F‐4500 fluorescence spectrofluorometer (Hitachi, Japan) using a quartz cell of 1.0 cm path length. The emission spectra were excited at 280 nm with a slit width of 5.0 nm and recorded between 300 and 400 nm. The measurement was obtained at a protein concentration of 0.2 mg/mL in PBS buffer (pH 7.4) at 25°C.

#### 2.6.5 TEM

TEM is a microscopy technique in which a beam of electrons is transmitted through a specimen to form an image and is often employed to evaluate the structure of protein samples [46]. In our study, 5 μL sample at concentration of 0.5 mg/mL was dipped and incubated the carbon‐Formvar coated copper grids (Zhongjingkeyi Technology, China) on the surface for 10 min and touched dry on blotting paper. Then, the grid was dipped and negatively stained with 1% uranyl acetate aqueous solution. The grids were examined according to the instructions of the JEM‐1400 TEM microscope (Hitachi, Japan), following the local guidelines.

#### 2.6.6 Immunization schemes

Female BALB/c mice aged at 6–8 weeks (body weight about 18–20 g) were purchased from SPF Biotechnology Co., Ltd, (Beijing, China) and maintained with pathogen‐free water and food. The animals were randomly divided into five groups for eight animals per group. The five groups were PBS (negative control) group, OVA (positive control) group, HBc VLP group, EBNA1‐HBc VLP group and HCV core‐HBc VLP group. HBc VLP, EBNA1‐HBc VLP, and HCV core‐HBc VLP samples were dialyzed against 20 mM PB, pH 7.4 before the immunization evaluation. For each group, the mice were immunized intraperitoneally with 100 μg of samples after filtered with 0.45 μm microfilter in 200 μL of sterile PBS on days 0, 14 (first boost), and 28 (second boost). For evaluation of the humoral immune response, immune serum was collected at day 24 and day 38 individually (10 days after first and second boost). Sera were isolated and stored at −70°C until use.

#### 2.6.7 ELISAs for antibody titer

Enzyme‐linked immunosorbent assays (ELISAs) were performed for the detection of epitope‐specific antibody titer. Briefly, HBc VLP and EBNA peptide (aa 407–417) and HCV core peptide (aa 10–53) were adsorbed overnight at 4°C to 96‐well plates at 10 μg/mL in 50 mM sodium carbonate buffer, pH 9.6. After being blocked with PBS containing 1% bovine serum albumin for 2 h at 37°C, serial dilutions of mouse sera were added to the plates and incubated for an additional 2 h at 37°C. After being washed 3 times with PBS containing 0.1% Tween‐20, an anti‐mouse IgG horseradish peroxidase‐conjugated antibody was applied at a 1:5000 dilution. Following 2 h of incubation at 37°C, the plates were washed, and 3,3’,5,5’‐tetramethylbenzidine single‐component substrate solution was added for color development. The color development was stopped by adding 1 M sulfuric acid. The results were checked in a microplate reader (Perlong, China) at 450 nm. The endpoint titers were defined as the highest serum dilution that resulted in an absorbance value two times greater than that of sera derived from mice in negative control group.

#### 2.6.8 Ethical statement

The *in vivo* study was approved by the Animal Ethics Committee of Shanxi University of Chinese Medicine (Shanxi, China, approval number: 2019LL137).

#### 2.6.9 MD simulation of chimeric HBc VLPs

To understand and investigate the possible causes for the differences of chimeric HBc VLPs after insertion of foreign epitopes, atomic structure of HBc (PDB ID: 3J2V) was employed. EBNA1 epitope (HPVGEADYFEY) from EBNA1 protein (PDB ID: 5WMF) and HCV core epitope (KTKRNTNRRPQDVKFPGGGQIVGGVYLLPRRGP RLGVRATRKTS) from HCV core protein (PDB ID: 1NLB) were added to the N‐terminus of HBc to form the atomic structure of chimeric EBNA1‐HBc and HCV core‐HBc using BIOVIA Discovery Studio. Hydrophobicity and 5‐residue running average hydrophobicity was calculated with the data from BIOVIA Discovery Studio. MD simulations were performed using GROMACS 2018.5 using GROMOS96 43a1 force field. SPC/E water model in a cubic box with a distance of 1.0 nm was applied for simulation and system was neutralized with Na and Cl ions. Energy minimization was performed using the steepest descent algorithm with a maximum force constraint of 1000.0 kJ/mol/nm. Particle Mesh Ewald (PME) method and Linear Constraint Solver (LINCS) algorithm was employed to calculate and constrain bonds. Temperature equilibration was performed at 300 K for 100 ps with the V‐rescale method. Then, the pressure was equilibrated with the pressure coupling of Parrinello–Rahman for 100 ps. Finally, MD simulations were run for 3 ns with 2‐fs time steps. The trajectories of the MD simulations were analyzed for RMSD and Rg. The RMSD and Rg data was visualized with Origin 9.

## 3 RESULTS AND DISCUSSION

### 3.1 Expression of chimeric HBc VLPs with insertion of foreign epitopes

**FIGURE 1:**
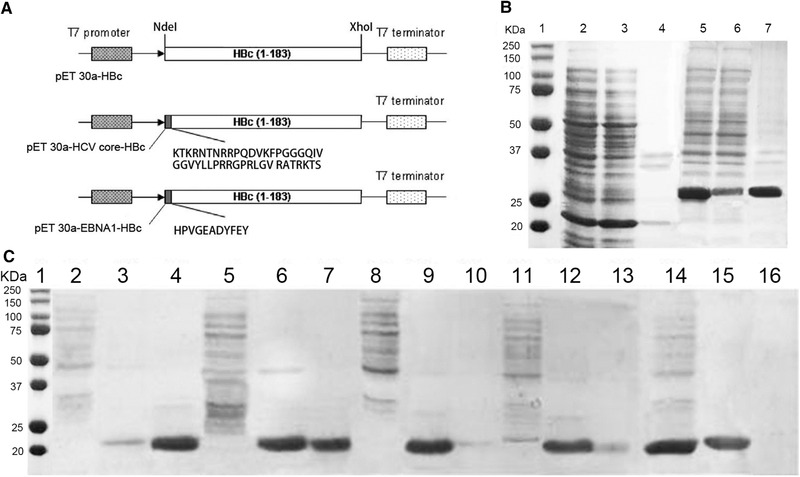
Schematic representation of the expression cassettes of the pET 30a‐HBc, pET 30a‐HCV core‐HBc, and pET 30a‐EBNA1‐HBc (A). SDS‐PAGE of expression of EBNA1‐HBc and HCV core‐HBc (B) and optimization of expression of HCV core‐HBc (C). (B) Lane 1: molecular weight marker; Lane 2: crude lysate of EBNA1‐HBc; Lane 3: supernatant of crude lysate of EBNA1‐HBc; Lane 4: pellets of crude lysate of EBNA1‐HBc; Lane 5: crude lysate of HCV core‐HBc; Lane 6: supernatant of crude lysate of HCV core‐HBc; Lane 7: pellets of crude lysate of HCV core‐HBc. (C) Lane 1: molecular weight marker; Lane 2,5,8, and 11: crude lysate of HCV core‐HBc; Lane 3,6,9, and 12: supernatant of crude lysate of HCV core‐HBc; Lane 4,7,10, and 13: pellets of crude lysate of HCV core‐HBc. (Condition 1: Lane 2–4, Condition 2: Lane 5–7, Condition 3: Lane 8–10, Condition 4: Lane 11–13)

As is illustrated in Figure 1A, long and structural epitope, HCV core epitope, and short and non‐structural EBNA1 were designed to be fused at N‐terminal of HBc to form the chimeric HCV core‐HBc VLP and EBNA1‐HBc VLP. pET 30a‐HCV core‐HBc plasmid was generated using Overlap Extension PCR [47]. Constructed gene sequence of HCV core‐HBc was confirmed by gene sequencing (data not shown). Then pET 30a‐HCV core‐HBc plasmid and pET 30a‐EBNA1‐HBc plasmid were transformed into *E. coli* BL21 (DE) strain for expression of HCV core‐HBc VLP and EBNA1‐HBc VLP proteins.

Regarding the expression of chimeric HBc VLP proteins, *E. coli* has been chosen because it is an efficient host cell to achieve high expression yield [48]. The expression condition of HCV core‐HBc and EBNA1‐HBc VLP proteins followed the similar conditions for the expression of HBc without epitope in previous work using 1 mM IPTG concentration, cell density OD_600_ = 1.2 for IPTG induction, and 37°C for expression temperature [39]. The concentration of target chimeric HBc VLP was estimated using ImageJ and the percentage of target soluble chimeric HBc VLP was calculated using Equation (1). As is shown in Figure 1B, around 97% target EBNA1‐HBc proteins were expressed in soluble form in the supernatant of bacteria lysate (Figure 1B, Lane 3) while only around 20% of HCV core‐HBc proteins were in soluble form in the supernatant of bacteria lysate (Figure 1B, Lane 6). The significant different percentages of soluble form expression of EBNA1‐HBc and HCV core‐HBc suggest that insertion of EBNA1 and HCV core epitopes had significant impact on the soluble expression of chimeric HBc VLPs in *E. coli* expression system. Insertion of short and non‐structural EBNA1 epitope has minor impact on the soluble expression; however, the insertion of long and structural HCV core epitope potentially increased the complexity for *E. coli* to fold HCV core‐HBc into the correct structure, therefore, more IBs were formed.

To reduce the formation of IBs of HCV core‐HBc and improve the soluble expression, single‐factor optimization of expression conditions including concentration of IPTG, expression temperature, and induction cell density was conducted orderly as is demonstrated in Table 1. As is shown in Figure 1C, SDS‐PAGE results for all four runs were merged for analysis. For induction cell density, the expressed soluble form of HCV core‐HBc proteins reached to 54% with lower induction cell density of OD_600_ at 0.8 while inducing at higher cell density (OD_600_ = 1.2), the soluble expression of HCV core‐HBc was 20%. For expression temperature evaluation, there is no significant difference of the percentage of soluble expressed HCV core‐HBc proteins between 37°C and 30°C. Therefore, 37°C was chosen as *E. coli* had a lower growth rate at 30°C, leading to less amount of biomass. For concentration of IPTG, more than 95% of HCV core‐HBc proteins were in the soluble form at the condition of 0.5 mM IPTG. Therefore, the optimal soluble expression conditions of HCV core‐HBc proteins were 0.5 mM IPTG was added at cell density of OD_600_ = 0.8 and 37°C for expression temperature.

**TABLE 1:** Optimization for expression of HCV core‐HBc

Condition	Concentration of IPTG	Temperature	Cell density (OD_600_)	Soluble target protein (%)
1	1 mM	37°C	1.2	20
2	1 mM	37°C	0.8	54
3	1 mM	30°C	0.8	52
4	0.5 mM	37°C	0.8	95

### 3.2 MD simulation to predict protein stability after insertion of different epitopes

**FIGURE 2:**
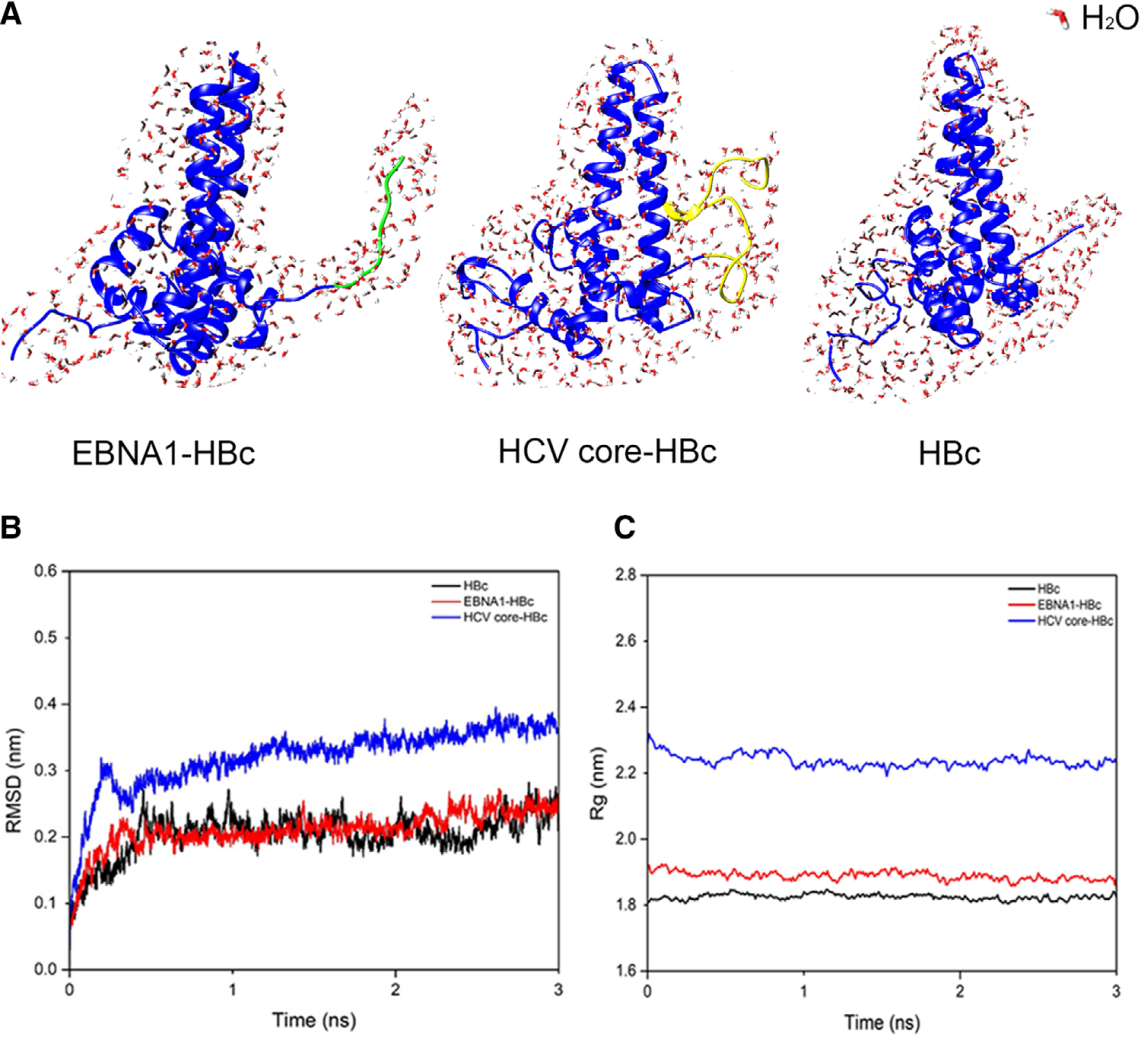
3D image of chimeric HBc VLPs in water environment (A). Plot of RMSD (B) and radius of gyration (C) of HBc, EBNA1‐HBc, and HCV core‐HBc monomer running with GROMACS in CHARMM force field in water environment. HBc in blue, EBNA1 in green, and HCV core in yellow

Insertion of foreign antigens to HBc VLP could have impacts on their stability, which would then influence the purification process. To predict the stability of chimeric HBc VLP after insertion of foreign epitopes and assist the design for purification process for removal of HCPs, MD simulation was applied to study the interaction between chimeric HBc VLP monomers and water molecule (Figure 2A). Data of RMSD and Rg of EBNA1‐HBc, HCV core‐HBc, and HBc was processed and plot into graphics. As is shown in Figure 2A, EBNA1‐HBc showed similar backbone RMSD compared with HBc while HCV core‐HBc had a higher RMSD, it indicates that HCV core‐HBc had a lower stability in water environment compared with EBNA1‐HBc and HBc. In Figure 2B, the Rg of HCV core‐HBc fluctuates about 0.2 nm higher than the fluctuation level of EBNA1‐HBc and HBc (less than 0.1 nm). The RMSD and Rg results indicated that HCV core‐HBc is less stable than other two samples in the water environment, which indicating that HCV core‐HBc VLP potentially had weaker interaction with water molecules in the environment. This finding potentially suggested that HCV core‐HBc was more hydrophobic, comparing with EBNA1‐HBc in theory.

### 3.3 Removal of HCPs by AS precipitation

**FIGURE 3:**
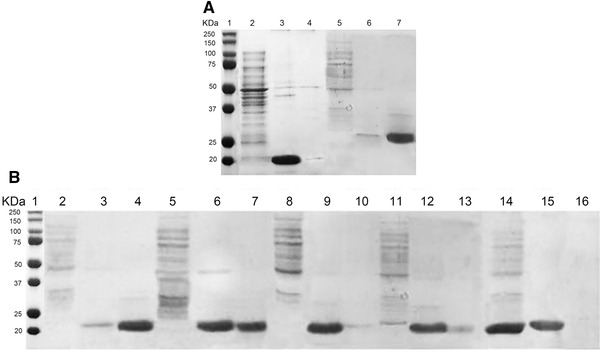
SDS‐PAGE of AS precipitation of chimeric HBc VLPs (A) and optimization of AS precipitation of HCV core‐HBc proteins (B). (A) Lane 1: molecular weight marker; Lane 2 and 5: supernatant after AS precipitation of EBNA1‐HBc and HCV core‐HBc; Lane 3 and 6: supernatant after resuspension of EBNA1‐HBc and HCV core‐HBc; Lane 4 and 7: pellet after resuspension of EBNA1‐HBc and HCV core‐HBc. (B) Lane 1: molecular weight marker; Lane 2, 5, 8, 11, and 14: supernatant after precipitation using 1, 0.5, 0.1, 0.05, and 0.01 M AS; Lane 3, 6, 9, 12, and 15: supernatant of resuspension using 4 M urea after precipitation using 1, 0.5, 0.1, 0.05, and 0.01 M AS; Lane 4, 7, 10,13, and 16: pellet of resuspension using 4 M urea after precipitation using 1, 0.5, 0.1, 0.05, and 0.01 M AS

HCPs are regarded as the major protein impurities in the production of biopharmaceuticals from intracellular expression process [49]. In our project, AS precipitation was employed to remove HCPs from expressed chimeric HBc VLP, because it is a low‐cost purification process in mild chemical condition. AS also has minor influence on the structure of proteins [3]. Following the AS precipitation conditions of HBc VLP in previous work, 1 M AS was used for precipitation and 4 M urea buffer was employed as the resuspension buffer for HCV core‐HBc VLP and EBNA1‐HBc VLP. As is illustrated in Figure 3A, the purity of resuspended EBNA1‐HBc reached 95% (Figure 3A, Lane 3) and the recovery yield for AS precipitation process was 93.35%, surprisingly, the recovery yield of HCV core‐HBc was only 5% with a purity of 97%. This result matches well with our previous finding in the MD simulation that HCV core‐HBc showed less stability in water environment and potentially more hydrophobic compared with EBNA1‐HBc, leading to its lower recovery yield.

Therefore, optimization for AS precipitation of HCV core‐HBc was conducted by using different concentrations of AS including 0.01, 0.05, 0.1, 0.5, and 1 M. The precipitated pellets were then resuspended using 4 M urea buffer. As is shown in Figure 3B, HCV core‐HBc proteins were fully precipitated with 1, 0.5, and 0.1 M AS from the lysate buffer (Figure 3B, Lane 2–10) while partial of HCV core‐HBc proteins was left in the supernatant of AS precipitation solution when using 0.05 and 0.01 M AS.

As is shown in Table S1, the highest recovery yield, 92.55%, was achieved by precipitating at 0.1 M AS with the purity of 96%. A lower recovery yield of 85.35% was obtained when precipitating by 0.05 M AS. The recovery yield achieved by other concentrations was relatively lower than that achieved by 0.1 M AS (5% for 1 M AS, 53.2% for 0.5 M AS, and 46.2% for 0.01 M AS). Higher concentrations of AS (1 and 0.5 M) could fully precipitate target HCV core‐HBc proteins; however, the formed precipitated protein samples by high concentrations of AS cannot be resuspended with 4 M urea buffer to achieve high yield (Figure 3B, Lane 4 and 7). This is possibly because high concentration of AS could lead to full dehydration of HCV core‐HBc thus resulting in irreversible denaturation or aggregation [50]. In contrast, although 0.01 M AS retained around 62% of target proteins in the supernatant of AS in the precipitation step (estimated using ImageJ), all target HCV core‐HBc proteins were resuspended using 4 M urea buffer (Figure 3B, Lane 14 and 15).

After AS precipitation, EBNA1‐HBc and HCV core‐HBc proteins was dialyzed against storage buffer (20 mM Tris‐HCl, pH 7.4) to remove 4 M urea buffer before the calculation of final production yield. Table S2 demonstrates the concentration and purity of target chimeric HBc VLPs after dialysis. The calculated final production yield of EBNA1‐HBc VLP was around 62.1 mg/g wet cell weight or 248.4 mg/L and final production yield of HCV core‐HBc protein was around 161.6 mg/L or 40.4 mg/g of wet cell weight.

It is worth to note that the achieved production yields of both HCV core‐HBc VLP and EBNA1‐HBc VLP are significantly higher than other reported HBc VLPs of 6.4 mg/L produced using cell‐free protein synthesis system [51] and 3.21  mg/L produced using *E. coli* expression system in IBs [52]. In addition, the achieved production yields are also higher than the reportedly high production yield in Pichia *pastoris* of 3 mg/g of wet cell weight [30].

### 3.4 Removal of host cell DNA by AEC

To remove host cell DNA impurities from produced chimeric HBc VLP proteins, POROS 50 HQ AEC was applied with a flow‐through strategy while DNA impurities are expected to bind with the resins and the target protein remains in the FT fractions [30]. Figure S1 illustrates the the chromatogram of POROS 50 HQ AEC, the amount of protein and DNA of chimeric HBc VLPs are presented in Table S3.

**FIGURE 4:**
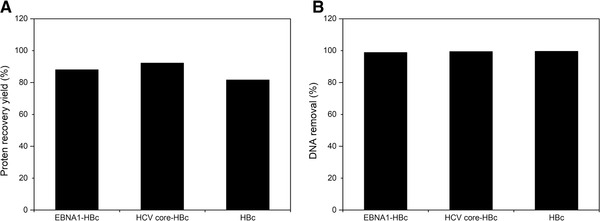
Percentages of protein recovery (A) and DNA removal (B) of EBNA1‐HBc, HCV core‐HBc, and HBc after POROS 50 HQ chromatography

Figure 4A,B illustrated the calculated percentage of protein recovery yield and DNA removal rate after POROS 50 HQ chromatography. Low percentages of DNA were detected in FT fraction of POROS 50 HQ chromatography of both chimeric HBc VLPs and HBc (EBNA1‐HBc: 1.09%, HCV core‐HBc: 0.57%, and HBc: 0.47%), while most target protein were detected in the FT fraction (EBNA1‐HBc VLP: 88.07%, HCV core‐HBc VLP: 92.23%, and HBc VLP: 81.63%). Around 99% of DNA removal from HBc, HCV core‐HBc, and EBNA1‐HBc was achieved by POROS 50 HQ chromatography. As is shown in Table S3, the residual DNA amount of EBNA1‐HBc, HCV core‐HBc, and HBc after purification was 0.06, 0.05, and 0.03 μg. However, A260/A280 ratio was still > 1.5 in the FT fraction of chromatography of all three cases (Figure S1). It was assumed that the higher A260/A280 ratio could be resulted from (1) HBc contains arginine‐rich domain (ARD) at C‐terminus that can capture RNA fragments [53]. (2) It is possible that RNA is buried inside chimeric HBc VLPs. This issue could be resolved by using further alkaline hydrolysis [11] or benzonase nuclease [39] to remove RNA. Or alternative method is to eliminate positive charged C‐terminal stretches [54] in the future molecule design.

### 3.5 Conformation of chimeric HBc VLPs after purification

Reports have indicated that the insertion of foreign epitopes to HBc VLP proteins can potentially decrease the stability of VLP structure or inhibit the assembly of HBc into VLP structure due to the nature of foreign epitopes [55, 56, 57]. To further evaluate whether the developed purification process is suitable to produce chimeric HBc VLPs in correctly assembled VLP structure, the purified EBNA1‐HBc VLP and HCV core‐HBc VLP were characterized using HPSEC, CD, IF, and TEM, the conformation of VLPs was compared with HBc VLP.

**FIGURE 5:**
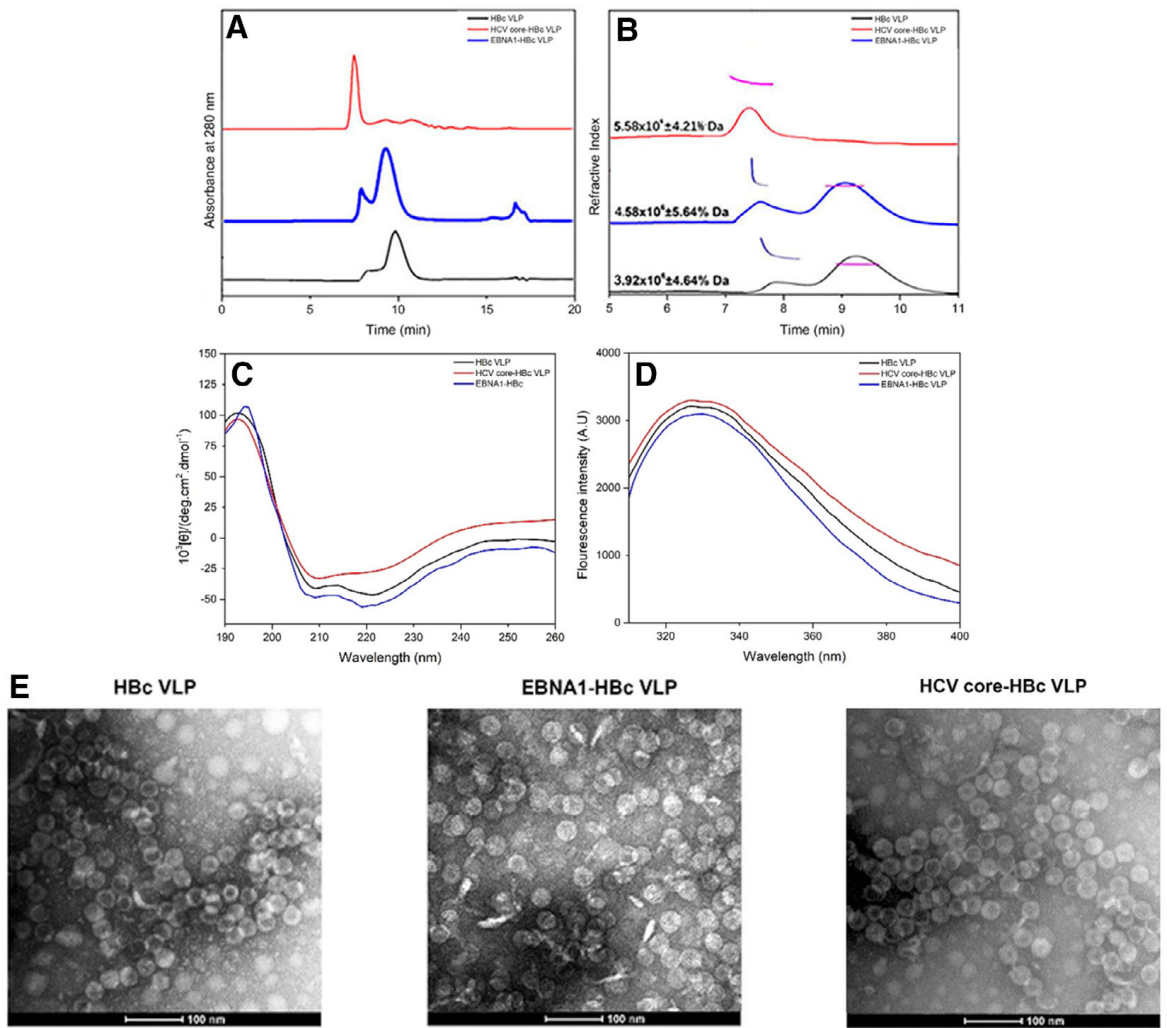
HPSEC (A)‐MALLS (B) of the purified EBNA1‐HBc and HCV core‐HBc on TSK G4000 SWXL column. Characterization of chimeric HBc VLP including CD spectra (C), IF spectra (D), and TEM analyses (E)

As is shown in Figure 5A,B, purified chimeric EBNA1‐HBc VLP showed similar morphology compared with HBc VLP. HPSEC‐MALLS is often used for the separation and analysis of protein sample according to their molecular weights [58, 59]. The molecular weight of chimeric EBNA1‐HBc VLP was around 4.58 × 10^6^ Da, which is close to the theoretical molecular weight of 3.97 × 10^6^ Da (T = 3) and 5.29 × 10^6^ Da (T = 4). The molecular weight of expressed HCV core‐HBc VLP was around 5.58 × 10^6^ Da, which is close to the theoretical molecular weight for HCV core‐HBc VLP (4.67 × 10^6^ Da [T = 3] or 6.23 × 10^6^ Da [T = 4]). This suggested that chimeric HBc VLPs were expressed in a mixture of T = 3 and T = 4 particles. Small amount of aggregation (around 15%) of both chimeric EBNA1‐HBc VLP and HBc VLP were observed in HPSEC‐MALLS results. However, in the HPSEC result, only one fraction peak was detected. This phenomenon could be explained by limitation of TSKgel G4000SWXL column that cannot separate between the aggregation peak and the VLP peak of HCV core‐HBc. The small amount of aggregation of chimeric HBc VLP could result from the aggregation behavior from HBc VLP due to the solution environment such pH and the buffering agent [60]. For example, Schumacher et al. claimed that self‐aggregation of HBc VLP was formed over time in Tris‐buffer [17].

The secondary structures of the HBc‐VLPs, chimeric EBNA1‐HBc VLP, and HCV core‐HBc VLP were examined by (CD) spectroscopy analysis. As is shown in Figure 5C, in comparison to HBc VLP, the peak at 222 nm, which represented the α‐helix structure of chimeric EBNA1‐HBc VLP, was negligible impacted after insertion of EBNA1 epitope. The increase of the ellipticity value at 222 nm of chimeric EBNA1‐HBc VLP suggests a structurally increase in α‐helix content after the insertion of foreign EBNA1 epitope to HBc VLP. On the other hand, HCV core‐HBc VLP had a large decrease of ellipticity value at peak of 222 nm in CD measurement, corresponding to the change of α‐helix structure. This suggests that the α‐helix structure on HCV core epitope could influence the formation of α‐helix in chimeric HCV core‐HBc, leading to the reduce of the ratio of α‐helix. In the fluorescent measurement of EBNA1‐HBc VLP, HCV core‐HBc VLP, and HBc VLP shown in Figure 5D, same fluorescence maximum emission wavelengths were observed. This suggests that there is no significant impact of the EBNA1 epitope and HCV core epitope on the tertiary structure of VLPs. The integrity of chimeric EBNA1‐HBc VLP and HCV core‐HBc VLP were characterized by TEM. TEM image in Figure 5E demonstrates that the produced EBNA1‐HBc and HCV core‐HBc proteins obtained the same VLP structure as HBc VLP.

The characterization results indicate that the insertion of long and structural foreign epitopes, HCV core, has a major impact on the secondary structure, but a minor impact on the tertiary structure and conformation. By contrast, EBNA1 made negligible impact on all levels of HBc VLP structure. The change on the secondary structure of HCV core‐HBc VLP, compared with EBNA1‐HBc VLP and HBc VLP, could explain the stability difference in MD simulation result. HCV core‐HBc was less stable in the water environment compared with EBNA1‐HBc and HBc. Aside this, the tertiary structure and TEM image results of HCV core‐HBc and EBNA1‐HBc proves that the developed purification process using AS precipitation and POROS 50 HQ chromatography has negligible impact on the assembly structure of both chimeric HBC VLPs, which could keep the antigenicity of both chimeric HBc VLPs.

### 3.6 Immunogenicity of chimeric HBc VLPs

**FIGURE 6:**
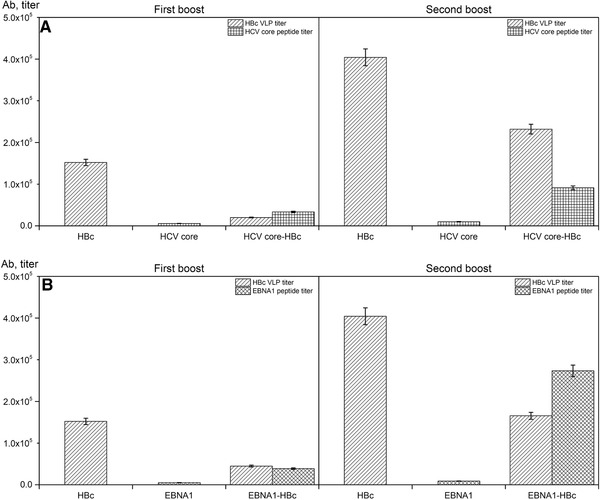
Production of antibodies (Ab) in BALB/c mice by chimeric HCV core‐HBc VLP (A) and EBNA1‐HBc VLPs (B). Shown are antibody titers from the sera collected 10 days after first boost and second boost. Titers in specimens were determined on plates coated with HBc VLP, EBNA1, and HCV core peptide as shown by different patterns

To evaluate the biological activity of purified EBNA1‐HBc and HCV core‐HBc VLPs, *in vivo* epitopic specific antibody titer level was measured. Female BALB/c mice were vaccinated with purified chimeric EBNA1‐HBc VLP and HCV core‐HBc VLP. Mice immunized with HBc VLP (without epitope), EBNA1 peptide, and HCV core peptide were used as reference groups. Two boosts of vaccination were performed at day 14 and day 28 after initial immunization. Serum of immunized mice after 10 days of first and second boosts (day 24 and day 38) was collected for the evaluation. As is shown in Figure 6A,B, both purified HCV core‐HBc and EBNA1‐HBc VLPs can induce epitopic specific antibody titer in the vaccinated mice compared with HBc VLP group. The epitopic specific antibody titer after second boost showed significant improvement compared with that of first boost (from less than 4 × 10^4^ for both chimeric HBc VLPs to 9.1 × 10^4^ and 2.7 × 10^5^ for HCV core‐HBc and EBNA1‐HBc VLP, respectively). The epitopic specific antibody titer induced by EBNA1 peptide group and HCV core peptide group was also measured; however, the level was relatively low compared with chimeric HBc VLP groups and HBc VLP group. This result demonstrates that two chimeric HBc VLPs are retained the correct assembled structure to stimulate the immune response against the inserted epitopes. In addition, we also found that HBc specific antibody titer was obviously detected in both chimeric HBc VLP groups; however, the total antibody titers of chimeric VLP vaccine groups were lower compared with that induced by HBc VLP. This could be resulted by the possible carrier‐induced epitopic suppression effect [61, 62].

## 4 CONCLUDING REMARKS

A cost‐effective production process was developed for two chimeric HBc VLPs presenting two different types of foreign epitopes, namely HCV core‐HBc VLP and EBNA1‐HBc VLP. After optimization of expression conditions, both HCV core‐HBc and EBNA1‐HBc proteins were successfully expressed in the soluble VLP form with high production yields that are 40.4 and 62.1 mg/g of wet cell weight, respectively. We found that control of expression rate is essential for expression of chimeric HBc protein in soluble form. In the aid of MD simulation of chimeric HBc VLPs, we found that after insertion of long and structural HCV core epitope, HCV core‐HBc was less stable in the water environment compared with EBNA1‐HBc and HBc without insertion of foreign epitopes. This finding guided the AS precipitation process for removal of HCPs. AS precipitation results indicate that 0.1 M AS was the optimal AS concentration for HCV core‐HBc, which has a recovery yield of 92.55% and purity of 96% and higher AS concentration causes the irreversible precipitation and low recovery yield. However, 1 M AS was the optimal AS concentration for EBNA1‐HBc with a recovery yield of 93.35% and purity of 95%. POROS 50 HQ chromatography was applied to remove host cell DNA and the DNA removal rates for both EBNA1‐HBc and HCV core‐HBc are more than 99% with protein recovery yield more than 88%. However, RNA fragments were still encapsidated because of the ARD at C‐terminus of HBc. Furthermore, we found that insertion of HCV core epitope had impact on the secondary structure of HCV core‐HBc, which potentially is the reason for the less stability of HCV core‐HBc, compared with EBNA1‐HBc and HBc. Aside this, the tertiary structure and TEM image results confirm that the produced chimeric HBc VLPs are in correctly assembled VLP structure. The immunogenicity evaluation of HCV core‐HBc and EBNA1‐HBc VLPs indicates that the achieved chimeric HBc VLPs are promising to obtain their biological activity as potential vaccine candidates against oncoviruses infection. Moreover, the developed cost‐effective production process has proved their advantages in the production of chimeric HBc VLPs presenting different foreign epitopes.

## CONFLICT OF INTEREST

The authors have declared no conflict of interest.

## Supporting information

Supporting Information

Supporting Information

## Data Availability

The data that support the findings of this study are available from the corresponding author upon reasonable request.

## References

[1] Ong, H.K. , Tan, W.S. and Ho, K.L. , Virus like particles as a platform for cancer vaccine development. Peer J. 2017, 5, 4053‐4053. 10.7717/peerj.4053 PMC5694210 29158984

[2] Buffin, S. , Peubez, I. , Barrière, F. , Nicolaï, M.‐C. , et al. Influenza A and B virus‐like particles produced in mammalian cells are highly immunogenic and induce functional antibodies. Vaccine 2019, 37, 6857–6867. 31590935 10.1016/j.vaccine.2019.09.057

[3] Kazaks, A. , Lu, I.N. , Farinelle, S. , Ramirez, A. , et al. Production and purification of chimeric HBc virus‐like particles carrying influenza virus LAH domain as vaccine candidates. BMC Biotechnol. 2017, 17, 79. 29126399 10.1186/s12896-017-0396-8 PMC5681787

[4] Kruger, S. , Ilmer, M. , Kobold, S. , Cadilha, B.L. , et al. Advances in cancer immunotherapy 2019 – latest trends. J Exp Clin Cancer Res. 2019, 38, 268. 31217020 10.1186/s13046-019-1266-0 PMC6585101

[5] Farrell, P.J. , Epstein–Barr virus and cancer. Annu. Rev. Pathol. 2019, 14, 29–53. 30125149 10.1146/annurev-pathmechdis-012418-013023

[6] Sander, A.F. and Lollini, P.L. , Virus‐like antigen display for cancer vaccine development, what is the potential? Expert Rev. Vaccines 2018, 17, 285–288. 29560746 10.1080/14760584.2018.1455505

[7] Pumpens, P and Grens, E. , HBV core particles as a carrier for B cell/T cell epitopes. Intervirology 2001, 44, 98–114. 11509871 10.1159/000050037

[8] Roose, K. , Baets, S.D. , Schepens, B and Saelens, X. , Hepatitis B core‐based virus‐like particles to present heterologous epitopes. Expert Rev. Vaccines 2013, 12, 183–198. 23414409 10.1586/erv.12.150

[9] Suffian, I.F.M. , Wang, J.T.W. , Faruqu, F.N. , Benitez, J. , et al. Engineering human epidermal growth receptor 2‐targeting hepatitis B virus core nanoparticles for siRNA delivery in vitro and in vivo. ACS Appl. Nano. Mater 2018, 1, 3269–3282. 30613831 10.1021/acsanm.8b00480 PMC6312360

[10] Suffian, M. , Wang, I. F. B. , Hodgins, J.T.‐W. , Klippstein, N. O. R. , et al. Engineering hepatitis B virus core particles for targeting HER2 receptors in vitro and in vivo. Biomaterials 2017, 120, 126–138. 28056402 10.1016/j.biomaterials.2016.12.012 PMC5300899

[11] Strods, A. , Ose, V. , Bogans, J. , Cielens, I. , et al. Preparation by alkaline treatment and detailed characterisation of empty hepatitis B virus core particles for vaccine and gene therapy applications. Sci Rep. 2015, 5, 11639. 26113394 10.1038/srep11639 PMC4650659

[12] Zhang, Y. , Song, S. , Liu, C. , Wang, Y. , et al. Generation of chimeric HBc proteins with epitopes in *E. coli*: Formation of virus‐like particles and a potent inducer of antigen‐specific cytotoxic immune response and anti‐tumor effect in vivo. Cell. Immunol. 2007, 247, 18–27. 17707782 10.1016/j.cellimm.2007.07.003

[13] Lei, Y. , Shao, J. , Zhao, F. , Li, Y. , et al. Artificially designed hepatitis B virus core particles composed of multiple epitopes of type A and O foot‐and‐mouth disease virus as a bivalent vaccine candidate. J. Med. Virol. 2019, 91, 2142–2152. 31347713 10.1002/jmv.25554

[14] Ye, X. , Ku, Z. , Liu, Q. , Wang, X. , et al. Chimeric virus‐like particle vaccines displaying conserved enterovirus 71 epitopes elicit protective neutralizing antibodies in mice through divergent mechanisms. J. Virol. 2014, 88, 72. 24131712 10.1128/JVI.01848-13 PMC3911748

[15] Peabody, D.S. , Manifold‐Wheeler, B. , Medford, A. , Jordan, S.K. , et al. Immunogenic display of diverse peptides on virus‐like particles of RNA phage MS2. J. Mol. Biol. 2008, 380, 252–263. 18508079 10.1016/j.jmb.2008.04.049 PMC2481506

[16] Caldeira, J.C. and Peabody, D.S. , Thermal stability of RNA phage virus‐like particles displaying foreign peptides. J. Nanobiotechnol. 2011, 9, 22. 10.1186/1477-3155-9-22 PMC3118325 21609437

[17] Schumacher, J. , Bacic, T. , Staritzbichler, R. , Daneschdar, M. , et al. Enhanced stability of a chimeric hepatitis B core antigen virus‐like‐particle (HBcAg‐VLP) by a C‐terminal linker‐hexahistidine‐peptide. J. Nanobiotechnol. 2018, 16, 39. 10.1186/s12951-018-0363-0 PMC5897928 29653575

[18] Huang, X. , Wang, X. , Zhang, J. , Xia, N . and Zhao, Q. , *Escherichia coli*‐derived virus‐like particles in vaccine development. NPJ. Vac. 2017, 2, 3. 10.1038/s41541-017-0006-8 PMC5627247 29263864

[19] Zeltins, A. , Construction and characterization of virus‐like particles: a review. Mol Biotechnol. 2013, 53, 92–107. 23001867 10.1007/s12033-012-9598-4 PMC7090963

[20] Ladd Effio, C. , Baumann, P. , Weigel, C. , Vormittag, P. , et al. High‐throughput process development of an alternative platform for the production of virus‐like particles in *Escherichia coli* . J. Biotechnol. 2016, 219, 7–19. 26707548 10.1016/j.jbiotec.2015.12.018

[21] Dai, W. , Xiong, P. , Zhang, X. , Liu, Z. , et al. Recombinant virus‐like particle presenting a newly identified coxsackievirus A10 neutralization epitope induces protective immunity in mice. Antiviral Res. 2019, 164, 139–146. 30817941 10.1016/j.antiviral.2019.02.016

[22] Sørensen, H.P. and Mortensen, K.K. , Soluble expression of recombinant proteins in the cytoplasm of *Escherichia coli* . Microb. Cell Fact. 2005, 4, 1. 15629064 10.1186/1475-2859-4-1 PMC544838

[23] Frietze, K.M. , Peabody, D.S and Chackerian, B. , Engineering virus‐like particles as vaccine platforms. Curr Opin Virol. 2016, 18, 44–49. 27039982 10.1016/j.coviro.2016.03.001 PMC4983494

[24] Linda, H.L , Lua, N.K.C. , Sainsbury, F. , Chuan, Y. P. , Wibowo, N. , Middelberg, A. P. J. , Bioengineering virus‐like particles as vaccines. Biotechnol. Bioeng. 2014, 111, 425–440. 24347238 10.1002/bit.25159

[25] Pereira Aguilar, P. , Schneider, T.A. , Wetter, V. , Maresch, D. , et al. Polymer‐grafted chromatography media for the purification of enveloped virus‐like particles, exemplified with HIV‐1 gag VLP. Vaccine 2019, 37, 7070–7080. 31300289 10.1016/j.vaccine.2019.07.001

[26] Ladd Effio, C. , Oelmeier, S.A. and Hubbuch, J. , High‐throughput characterization of virus‐like particles by interlaced size‐exclusion chromatography. Vaccine 2016, 34, 1259–1267. 26845741 10.1016/j.vaccine.2016.01.035

[27] Li, Z. , Wei, J. , Yang, Y. , Ma, X. , et al. Strong hydrophobicity enables efficient purification of HBc VLPs displaying various antigen epitopes through hydrophobic interaction chromatography. Biochem. Eng. J. 2018, 140, 157–167.

[28] Shaddeau, A.W. , Schneck, N.A. , Li, Y. , Ivleva, V.B. , et al. Development of a new tandem ion exchange and size exclusion chromatography method to monitor vaccine particle titer in cell culture media. Anal. Chem. 2019, 91, 6430–6434. 31034206 10.1021/acs.analchem.9b00095 PMC11040568

[29] Yang, Y. , Mengran, Y. , Zhang, S. , Ma, G and Su, Z. , Adsorption of virus‐like particles on ion exchange surface: Conformational changes at different pH detected by dual polarization interferometry. J. Chromatogr. A 2015, 1408, 161–168. 26189208 10.1016/j.chroma.2015.07.019

[30] Freivalds, J. , Dislers, A. , Ose, V. , Pumpens, P. , et al. Highly efficient production of phosphorylated hepatitis B core particles in yeast *Pichia pastoris* . Protein Expr. Purif. 2011, 75, 218–224. 20854910 10.1016/j.pep.2010.09.010

[31] Zahin, M. , Joh, J. , Khanal, S. , Husk, A. , et al. Scalable production of HPV16 L1 protein and VLPs from tobacco leaves. PLoS One 2016, 11, 160995. 10.1371/journal.pone.0160995 PMC4982596 27518899

[32] Duong‐Ly, K.C. and Gabelli, S.B. , Chapter seven ‐ salting out of proteins using ammonium sulfate precipitation, In: J. Lorsch, ed. Methods in Enzymology. Academic Press; 2014:85‐94. 10.1016/B978-0-12-420119-4.00007-0 24674064

[33] Kazaks, A. , Lu, I.N. , Farinelle, S. , Ramirez, A. , et al. Production and purification of chimeric HBc virus‐like particles carrying influenza virus LAH domain as vaccine candidates. BMC Biotechnol. 2017, 17, 79‐79. 29126399 10.1186/s12896-017-0396-8 PMC5681787

[34] Iyer, G. , Ramaswamy, S. , Cheng, K.‐S. , Sisowath, N. , et al. Flow‐through purification of viruses‐ a novel approach to vaccine purification. Procedia Vaccinol. 2012, 6, 106–112.

[35] Parra, S.C. and GebSki, C. , Benefits of a Revised Approach to Anion Exchange Flow‐Through Polish Chromatography A highperformance anion exchange resin performs well compared with membranes. In addition, the resin offers greater flexibility and cost savings. BioPharm Int. 2011, 24, S1–S5.

[36] Gerstweiler, L. , Bi, J. and Middelberg, A. , Virus‐like particle preparation is improved by control over capsomere‐DNA interactions during chromatographic purification. Biotechnol. Bioeng. 2011.118 4:1688–1701. 10.1002/bit.27687 33484156

[37] Mobini, S. , Chizari, M. , Mafakher, L. , Rismani, E and Rismani, E. , Computational design of a novel VLP‐based vaccine for hepatitis B virus. Front Immunol. 2020, 11:2074. 33042118 10.3389/fimmu.2020.02074 PMC7521014

[38] Zhang, L. , Tang, R. , Bai, S. , Connors, N.K. , et al. Energetic changes caused by antigenic module insertion in a virus‐like particle revealed by experiment and molecular dynamics simulations. PLoS One 2014, 9, 107313. 10.1371/journal.pone.0107313 PMC4162605 25215874

[39] Zhang, Y. , Liu, Y. , Zhang, B. , Yin, S. , et al. In vitro preparation of uniform and nucleic acid free hepatitis B core particles through an optimized disassembly‐purification‐reassembly process. Protein Expr. Purif. 2020, 178:105747. 32898688 10.1016/j.pep.2020.105747

[40] Bradford, M.M. , A rapid and sensitive method for the quantitation of microgram quantities of protein utilizing the principle of protein‐dye binding. Anal. Biochem. 1976, 72, 248–254. 942051 10.1016/0003-2697(76)90527-3

[41] Schneider, C.A. , Rasband, W.S. and Eliceiri, K.W. , NIH Image to ImageJ: 25 years of image analysis. Nat. Methods 2012, 9, 671–675. 22930834 10.1038/nmeth.2089 PMC5554542

[42] Gupta, R.B. and Shepherd, K. , Two‐step one‐dimensional SDS‐PAGE analysis of LMW subunits of glutelin. Theor. Appl. Genet. 1990, 80, 65–74. 24220812 10.1007/BF00224017

[43] Greenfield, N.J. , Using circular dichroism spectra to estimate protein secondary structure. Nat Protoc. 2006, 1, 2876–2890. 17406547 10.1038/nprot.2006.202 PMC2728378

[44] Gorinstein, S. , Goshev, I. , Moncheva, S. , Zemser, M. , et al. Intrinsic tryptophan fluorescence of human serum proteins and related conformational changes. Journal of Protein Chemistry 2000, 19, 637–642. 11307947 10.1023/a:1007192017291

[45] Vivian, J.T. and Callis, P.R. , Mechanisms of tryptophan fluorescence shifts in proteins. Biophy J. 2001, 80, 2093–2109. 10.1016/S0006-3495(01)76183-8 PMC1301402 11325713

[46] Arakawa, H. , Umemura, K and Ikai, A. , Protein images obtained by STM, AFM and TEM. Nature 1992, 358, 171–173. 1377369 10.1038/358171a0

[47] Bryksin, A.V. and Matsumura, I. , Overlap extension PCR cloning: a simple and reliable way to create recombinant plasmids. BioTechniques 2010, 48, 463–465. 20569222 10.2144/000113418 PMC3121328

[48] Xiao, Y. , Chen, H.Y. , Wang, Y. , Yin, B. , et al. Large‐scale production of foot‐and‐mouth disease virus (serotype Asia1) VLP vaccine in *Escherichia coli* and protection potency evaluation in cattle. BMC Biotechnol. 2016, 16, 56. 27371162 10.1186/s12896-016-0285-6 PMC4930597

[49] Vanderlaan, M. , Zhu‐Shimoni, J. , Lin, S. , Gunawan, F. , et al. Experience with host cell protein impurities in biopharmaceuticals. Biotechnol Prog. 2018, 34, 828–837. 29693803 10.1002/btpr.2640

[50] Simpson, R.J. , Fractional precipitation of proteins by ammonium sulfate. Cold Spring Harb Protoc. 2006, 2006, 4309. 10.1101/pdb.prot4309 22485685

[51] Spice, A.J. , Aw, R. , Bracewell, D.G. and Polizzi, K.M. , Synthesis and assembly of hepatitis B virus‐like particles in a *Pichia pastoris* cell‐free system. Front Bioeng Biotechnol. 2020, 8:72. 32117947 10.3389/fbioe.2020.00072 PMC7033515

[52] Bin Mohamed Suffian, I.F. , Garcia‐Maya, M. , Brown, P. , Bui, T. , et al. Yield optimisation of hepatitis B virus core particles in *E. coli* expression system for drug delivery applications. Sci Rep. 2017, 7, 43160. 28256592 10.1038/srep43160 PMC5335696

[53] Chu, T.‐H. , Liou, A.‐T. , Su, P.‐Y. , Wu, H.‐N and Shih, C. , Nucleic acid chaperone activity associated with the arginine‐rich domain of human hepatitis B virus core protein. J Virol. 2014, 88, 2530–2543. 24352445 10.1128/JVI.03235-13 PMC3958103

[54] Dishlers, A. , Skrastina, D. , Renhofa, R. , Petrovskis, I. , et al. The hepatitis B virus core variants that expose foreign C‐terminal insertions on the outer surface of virus‐like particles. Mol Biotechnol. 2015, 57, 1038–1049. 26446016 10.1007/s12033-015-9895-9 PMC4619458

[55] Karpenko, L. , Ivanisenko, V. , Pika, I. , Chikaev, N. , et al. Insertion of foreign epitopes in HBcAg: how to make the chimeric particle assemble. Amino Acids 2000, 18, 329–337. 10949916 10.1007/s007260070072

[56] Vogel, M. , Vorreiter, J and Nassal, M. , Quaternary structure is critical for protein display on capsid‐like particles (CLPs): efficient generation of hepatitis B virus CLPs presenting monomeric but not dimeric and tetrameric fluorescent proteins. Proteins 2005, 58, 478–488. 15526302 10.1002/prot.20312

[57] Blokhina, E.A. , Kuprianov, V.V. , Stepanova, L.A. , Tsybalova, L.M. , et al. A molecular assembly system for presentation of antigens on the surface of HBc virus‐like particles. Virology 2013, 435, 293–300. 23062739 10.1016/j.virol.2012.09.014

[58] Tennikova, T. , Svec, F and Belenkii, B. , High‐performance membrane chromatography. A novel method of protein separation. J. Liq. Chromatogr. 1990, 13, 63–70.

[59] Oliva, A. , Llabrés, M and Fariña, J.B. , Comparative study of protein molecular weights by size‐exclusion chromatography and laser‐light scattering. J. Pharm. Biomed. Anal. 2001, 25, 833–841. 11377066 10.1016/s0731-7085(01)00359-4

[60] Chen, Y. , Zhang, Y. , Quan, C. , Luo, J. , et al. Aggregation and antigenicity of virus like particle in salt solution—a case study with hepatitis B surface antigen. Vaccine 2015, 33:4300‐4306. 25862298 10.1016/j.vaccine.2015.03.078

[61] Schutze, M.P. , Leclerc, C. , Jolivet, M. , Audibert, F . and Chedid, L. , Carrier‐induced epitopic suppression, a major issue for future synthetic vaccines. J. Immunol. 1985, 135, 2319–2322. 2411793

[62] Jegerlehner, A. , Wiesel, M. , Dietmeier, K. , Zabel, F. , et al. Carrier induced epitopic suppression of antibody responses induced by virus‐like particles is a dynamic phenomenon caused by carrier‐specific antibodies. Vaccine 2010, 28, 5503–5512. 20307591 10.1016/j.vaccine.2010.02.103

