# Supplementary figures and images for: Cost‐effective purification process development for chimeric hepatitis B core (HBc) virus‐like particles assisted by molecular dynamic simulation
Source: Eng Life Sci. 2021 May 3;21(6):438–52. doi: 10.1002/elsc.202000104 (PMC8182290; doi:10.1002/elsc.202000104)

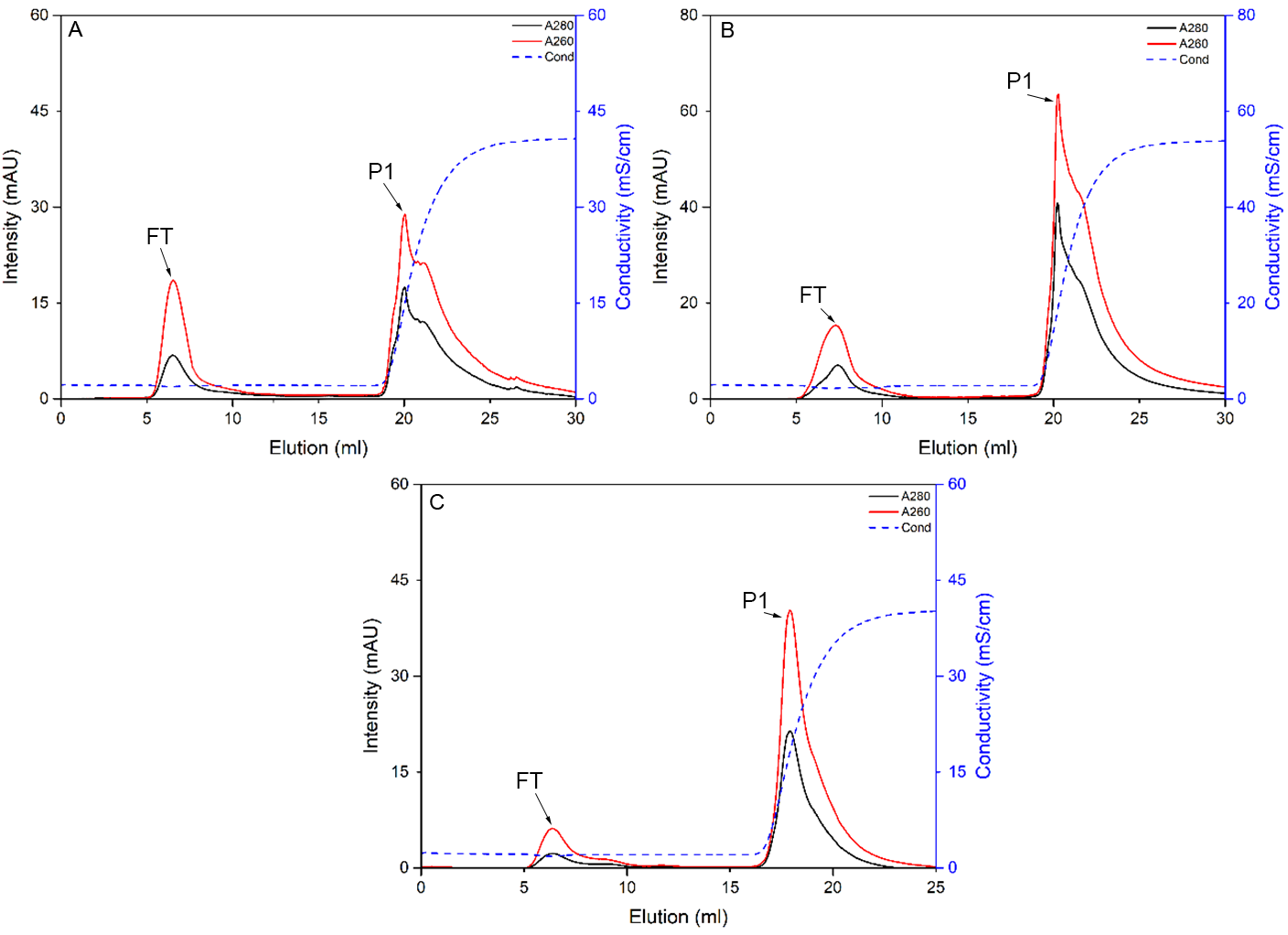

Supplement: Supplementary file 1 — Supporting Information [file ELSC-21-438-s002.tif]
