# Supplementary material for: Cost‐effective purification process development for chimeric hepatitis B core (HBc) virus‐like particles assisted by molecular dynamic simulation
Source: Eng Life Sci. 2021 May 3;21(6):438–52. doi: 10.1002/elsc.202000104 (PMC8182290; doi:10.1002/elsc.202000104)
Supplement: Supplementary file 2 — Supporting Information [file ELSC-21-438-s001.pdf]

Supporting information

Cost-effective purification process development for chimeric Hepatitis B core (HBc) virus like particles assisted by molecular dynamic simulation

Bingyang Zhang[1],

Shuang Yin[1],

Yingli Wang[2]

Zhiguo Su[3],

Jingxiu Bi[1].

1. School of Chemical Engineering & Advanced Materials, Faculty of Engineering, Computer and Mathematical Sciences, University of Adelaide, Adelaide, Australia

2. School of Chinese Medicine and Food Engineering. Shanxi University of Traditional Chinese Medicine .121 High school road, Yuci district, Jinzhong, Shanxi Province, 030619, China

3. State Key Laboratory of Biochemistry Engineering, Institute of Process Engineering, Chinese Academy of Sciences, Beijing, China

Correspondence: A/Prof Jingxiu Bi (jingxiu.bi@adelaide.edu.au). School of Chemical Engineering &Advanced Materials, Faculty of Engineering, Computer and Mathematical Sciences, University of Adelaide, North terrace, SA5005, Adelaide, Australia.

Figure S1. POROS™ 50 HQ chromatography of chimeric HBc VLPs using 20 mM Tris-HCl, pH 9 as equilibration buffer and 20 mM Tris-HCl, pH 9 with 1 M NaCl as elution buffer.

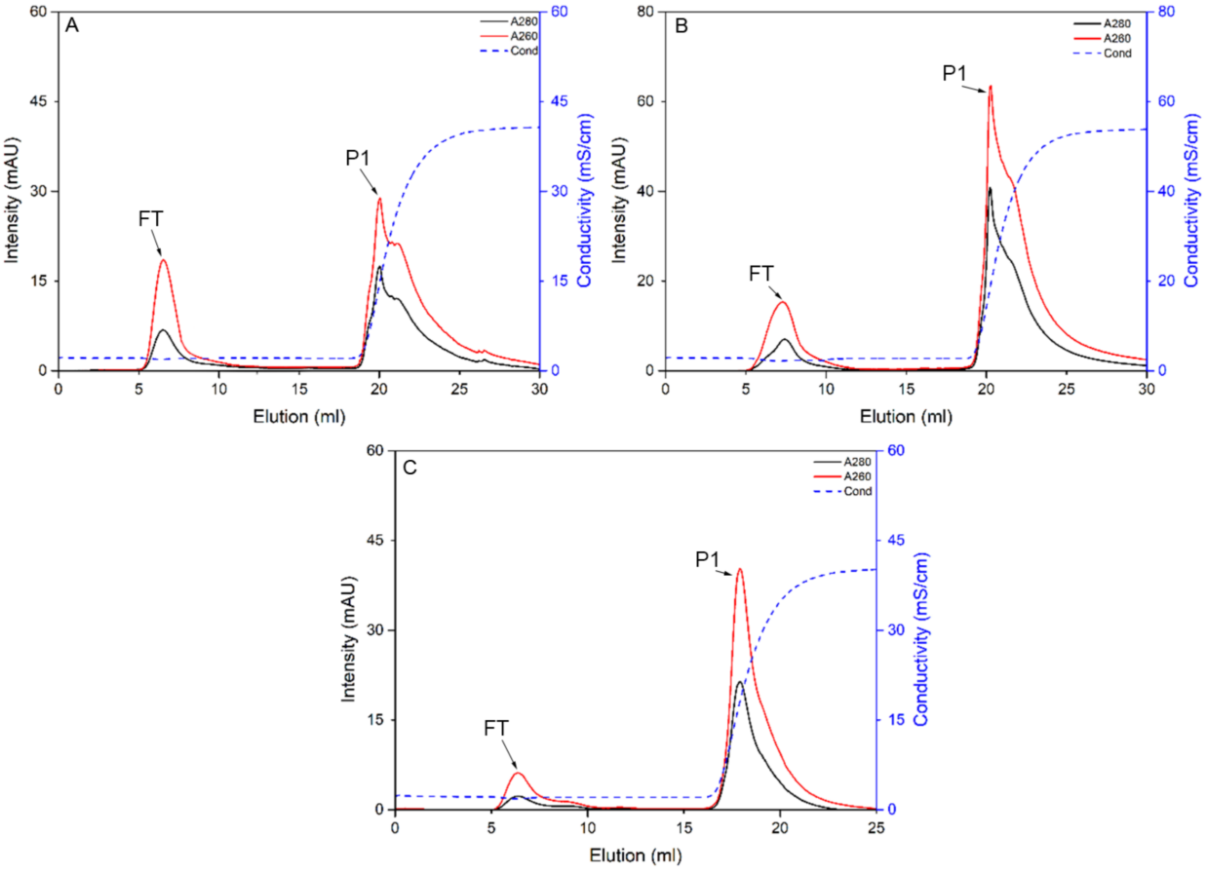

A: Chromatography result of EBNA1-HBc. **B**: Chromatography result of HCV core-HBc. **C**, Chromatography result of HBc. FT: flow-through fraction, P1: elution fraction

Table S1. AS precipitation recovery yields of HCV core-HBc precipitated with different concentrations of AS.

No.	Concentration of AS	AS precipitation Recovery yield of HCV core-HBc	Purity of recovered HCV core-HBc
**1**	1 M	5 %	98 %
**2**	0.5 M	53.2 %	95 %
**3**	0.1 M	92.55 %	96 %
**4**	0.05 M	85.35 %	94 %
**5**	0.01 M	46.2 %	99 %

Table S2. Recovery yield and purity of chimeric HBc VLPs after dialysis against storage buffer.

VLP	Concentration before dialysis (mg/ml)	Concentration after dialysis (mg/ml)	Recovery Yield	Purity after dialysis
**EBNA1-HBc**	1.05	1.02	96.17 %	99 %
**HCV core-HBc**	1.12	1.01	88.28 %	93 %

Table S3 Protein and nucleic acid mass in POROS™ 50 HQ chromatography of chimeric HBc VLPs and HBc VLP.

Sample/Mass	Before loading		FT fraction		P1 fraction	
**Total**	DNA (μg)	Target Protein (mg)	DNA (μg)	Target Protein (mg)	DNA (μg)	Target Protein (mg)
**EBNA1-HBc**	5.07	1.09	0.06	0.96	4.88	0.09
**HCV core-HBc**	9.31	1.03	0.05	0.95	8.85	0.15
**HBc**	7.29	0.98	0.03	0.80	5.91	0.15
